# A Review of the Tuberculosis Problem1Presidential address at the thirtieth annual meeting of the Alumni Association of the Medical Department, University of Buffalo, May 3, 1905.

**Published:** 1905-06

**Authors:** DeLancey Rochester

**Affiliations:** Buffalo. N. Y. Associate Professor of Medicine, University of Buffalo


### BUFFALO MEDICAL JOURNAL.

VOL. XLIV.—LX. JUNE, 1905. NO. 11

### ORIGINAL COMMUNICATIONS.

### A Review of the Tuberculosis Problem.[1]

BY DELANCEY ROCHESTER, M. D., Buffalo, N. Y.
Associate Professor of Medicine, University of Buffalo.

I PROPOSE this evening to review briefly the advances that have been made in internal medicine during the past year, and to conclude with a rather more extended review of the tuberculosis problem, presenting my own views as to the most important factor in the prevention of the spread of this disease.

Before, however, proceeding to this review, I want to call your attention to the fact of the utter impossibility of practising medicine at the present day without a microscope and the ability to use it. Anyone who attempts to do so is not acting fairly to himself or his patients. Every year I try to impress upon the students that the first investment they make should be, not in obstetric forceps, or a set of surgical instruments, but in a microscope and a complete urinalysis outfit. The value of this advice is shown in the

REVIEW OF PROGRESS.

In typhoid fever, the importance of infection by contact has been brought prominently forward by several observers, as a means of dissemination of the disease, to which sufficient attention has not been directed in the past, and the ease by which it may be avoided by the simple means of frequent thorough washing of the hands of attendants. A number of instances of carrying the infection by means of centrifugalised cream and butter, have been reported. Attention is called by several observers to

1. Presidential address at the thirtieth annual meeting of the Alumni Association of the Medical Department, University of Buffalo, May 3, 1905.

the greater frequency of hemorrhages, neuritis and phlebitis in those cases treated by the Brand bath. When, however, we realise the lower death rate obtained by this method, it is not surprising that there should be an increase in the complications and sequelæ. A number of observations by different investigators have shown almost invariably a rise of blood pressure in case of perforation with peritonitis, coincident with, and sometimes preceding, the pain, and always before the symptoms of collapse. Further observations are, however, necessary before formulating a positive rule.

Nothing new has been added to our knowledge of pneumonia and pleurisy, diagnostically or therapeutically, except that there has been a decided increase in the number of reported cases ushered in or accompanied by severe abdominal pain.

In diphtheria the profession generally is learning to recognise the value of the early bacteriological diagnosis and prompt administration of large doses of antitoxin. Perhaps the most noteworthy advances in relation to diphtheria, have been in the recognition of the value of the administration of the prophylactic dose to all other members of a household in which a case of diphtheria occurs. My own experience in the use of diphtheria antitoxin is that since beginning its use I have not lost a case in private practice; and in hospital practice only those cases that have come in too late for anything to be of any avail. Moreover, in private practice, by the prompt use of the prophylactic dose, I have prevented the development of any new case in a family where a case has been discovered. This is the experience of all who use this remedy sufficiently early and in sufficiently large dose. If I were so situated that I could not avail myself of bacterial diagnosis, to every doubtful case I should give antitoxin. I have given enormous doses,—10,000 units at a single dose, and as much as 20,000 units in 24 hours, and have never seen any evil results beyond an evanescent urticaria, or a few joint pains, which readily yielded to a purge, alkalies, and occasionally small doses of sodium salicylate or aspirin. A. Caillé proposes the regular immunisation of school children two or three times a year during the school year, by the injection of the prophylactic dose, 200 to 500 units,—as a means of preventing diphtheria and the diphtheritic complication of scarlet fever and measles,—and gives statistics showing the value of such procedure.

Of other sera used for therapeutic purposes, the only one that is of proven value is the antitetanus serum, though evidence is accumulating that seems to give some reason for hope that before long we will have a reliable serum with which to control the streptococcus. Antiplague, anticholera and antidysentery sera are apparently more useful as immunising than as therapeutic agents.

Cerebrospinal meningitis has of late attracted great attention, on account of the severe epidemics raging in different parts of the world, which give the disease almost a pandemic character. The cause is now accepted as the diplococcus intracellularis of Weichselbaum; but therapeutically the only advance that has been made is the practice of lumbar puncture and withdrawal of the cerebrospinal fluid. In several instances this procedure has apparently been the means of saving life, but it is by no means the cure-all that some zealous advocates would have us believe. Continuous drainage of the cerebrospinal canal by this means, under thorough asepsis, is advocated, but has not been given sufficient trial.

The rôle of protozoa as producers of disease is prominently before the profession, especially through the work of Councilman and Calkins in regard to smallpox, and inferentially of the whole class of the exanthemata; and through the work of Plimmer, Clewes, Gaylord and Calkins in relation to the etiology of cancer. The conclusions of all these observers are still subjudice, awaiting confirmation.

In the etiology of arteriosclerosis, through the conscientious work of Cabot and others, alcohol has almost been ruled out as a factor of any great importance; and through the observations of Thayer and others the acute infections, especially typhoid, malaria and influenza, are assuming greater importance.

The importance of autointoxication from the mouth and from the colon, and the necessity of thorough cleansing of both ends of the digestive tract, are becoming more generally recognised.

To Stockton, clinically, and to Charlton, of Montreal, experimentally, we owe the knowledge that infection by the colon bacillus of low virulence will produce an anemia which is almost the counterpart of severe pernicious anemia, and unquestionably some cases in the past of anemia, tabulated as pernicious, must have been due to such colon bacillus infection.

The importance of chronic nephritis as an etiological factor in many disturbances of the metabolism, especially such as are often classed under the heading of asthenia or of neurasthenia, is being more generally recognised, and necessity for frequent examination of the total twenty-four hours excretion of urine in all such cases is paramount. In the majority of such cases, the microscope gives us the most important information of any one procedure in the examination.

In the treatment of nephritis, Bendix has demonstrated clearly that it is possible for the skin to take up vicariously the action of kidneys, thus proving the scientific value of the well recognised clinical procedure of induced sweating.

Widal and Javal have proven the value of salt starvation in the reduction of the dropsies of nephritis, and that a patient with chronic parenchymatous nephritis may be allowed a fairly free diet, including bread, meat, sugar, potato, butter, and the like, provided that the foods are not salted.

### THE TUBERCULOSIS PROBLEM.

It is impracticable for me to undertake to review the advances in surgery, gynecology, obstetrics and the minor specialties, so I shall proceed immediately to a brief discussion of the tuberculosis problem as it now presents itself to us.

As to the prime etiological factor, the profession is now practically unanimous in giving that rôle to Koch's bacillus; and the existence of tuberculosis in an ancestor, unless the patient has been more or less constantly associated with that ancestor, is no longer considered of any etiological significance. Therefore, the study of the environment of the patient, especially the presence of other tubercular cases in the home, or the school, or the factory, or other place of work or residence, assumes the greater etiological importance.

The rôle of bovine tuberculosis as an etiological factor of the greatest importance, is still insisted upon by von Behring, who maintains that human pulmonary consumption is the end-stage of an infection acquired in infancy. Most observers do not agree with Behring, the concensus of opinion being that bovine tuberculosis is a factor in the etiology of human tuberculosis, but a relatively small one; that, nevertheless, all efforts should be maintained to suppress it. Of interest in this connection are the observations of Naegeli in the postmortem room at Zurich. He found practically everyone above 30 to be tuberculous, between 18 and 30, about 96 per cent. had traces of the disease; 50 per cent. of those between 14 and 18; 33 per cent. of those between 5 and 14; and 17 per cent. between 1 and 5, had tuberculous foci. *Infants less than 12 months old were usually found uninfected.*

Franz, of Budapest, tried the tuberculin reaction in soldiers, in doses of 1 to 5 mgm. In the first year, 61 per cent. were tuberculous; in the second year, 68 per cent. reacted positively; when 10 mgm. were used, 96 per cent. reacted. Of 96 infants in whom Behrend employed the same dose, all gave a negative result.

The observations of Harbitz, reported in the *Journal of Infectious Diseases,* for March 1, 1905, go far to support the contention of Behring that tuberculosis is primarily a lymphnode infection by way of the throat or intestinal tract, and that the pulmonary disease is a later manifestation of the disease.

From the observations of most investigators, the largest number of cases of tuberculosis in children occur during the second and third year; and this is readily explained in two ways—namely, infection through food, including milk, and infection from the floors, walls and furniture of infected surroundings,—children of this age crawling about on the floors and putting their hands and any article they may pick up into their mouths.

Whether the route of infection is most commonly by the digestive or the respiratory tract, is of secondary importance, in my opinion, to the well established fact that the chief, if not the only, source of infection is the sputum of the subjects of pulmonary tuberculosis. The discharge from other tubercular lesions is relatively slight, and is almost invariably carefully destroyed. It is possible that people dead of tuberculosis and buried, may infect the soil. Tubercle bacilli have been found in earth worms.

Patients with advanced consumption throw off in the expectoration countless millions of the bacilli daily. Some idea of the extraordinary number may be gained from the studies of Nuttall. From a patient with moderately advanced disease, the amount of whose expectoration was from 70 to 130 cc. daily (a quarter to half a tumblerful), he estimated that there were in sixteen counts, between January 10 and March 1, from 1½ to 4⅓ billions of bacilli thrown off in the 24 hours. . . .
The observations made by Cornet under Koch's supervision, are, in this connection, most instructive. He collected dust from the walls and bedsteads of various localities, and determined its virulence or innocuousness by inoculation with susceptible animals. . . . Of 118 dust samples from hospital wards or the rooms of consumptive patients, 40 were infectious and produced tuberculosis. . . . In a room in which a tuberculous woman had lived, the dust from the wall in the neighborhood of the bed was infective six weeks after her death.

In the Paris General Hospitals, tuberculosis decimates the attendants. On the other hand, the statistics of the Brompton Consumption Hospital, of Detweiler's Sanatorium at Falkenstein, and all thoroughly equipped and carefully regulated hospitals devoted to the care of consumptives, in which the sputum is collected and destroyed, nurses and attendants are rarely attacked.

It is interesting to know that by exposure to the direct rays of the sun, the tubercle bacilli is killed in a few hours, and, exposed to diffused sunlight, it is killed in one to six days. . . . but moisture, darkness and bad air can keep them alive for weeks. In dark places there is the greatest danger of infection, and narrow alleys, courts and dwellings shut off from light always have been rightly regarded as the special breeding-places of consumption.[1]

The careful compilation of statistics for a great many years, in many places, shows that tuberculosis causes one-seventh of all deaths, and that tuberculosis of the lungs—consumption—causes one-tenth of all deaths: that it works its greatest havoc in early middle life: according to the statistics of Lillian Brandt, of the New York C. O. S., it causes one-third of all the deaths that occur between the ages of fifteen and forty-four.

The following most valuable contribution to the economics of tuberculosis is made by Cornet in his exhaustive treatise on the subject of Tuberculosis, in Nothnagel's Encyclopedia:

Averaging the mortality reports for the years 1876 to 1891, in Prussia, the annual mortality from tuberculosis in the class of breadwinners, including the ages of fifteen to seventy, was 71,895, or a third of the entire number of deaths. Consumption runs a number of years before it proves fatal; but, if we reckon the invalidism due to it at only one year and the average loss of wage (or unemployed labor) at a value of two marks per day, then the loss of earnings for each person per year, 300 working days to the year, amount to 600 marks; so, for the total number of those who have died in the breadwinning period, 71,895 persons, the annual loss of earnings amount to 43,137,000 marks annually.

If we add to this figure, the money for doctors and medicine, for food and care (counting that the sick pay out 2.19 marks per day), for burial, and furthermore, the expenditure for invalids not included among breadwinners, we may estimate at least twice as much as the previous figure, or 86 million marks as the yearly cost of tuberculosis to the Prussian state.

A similar estimation of the cost to the state has been made by Dr. Herman M. Biggs, of New York, giving as a total annual loss to the city from tubercular diseases of at least 23 million dollars, and to the United States of more than 330 million dollars.

By looking a little more closely at our subject, we find that in certain occupations the disease is more prevalent and more fatal than in others. Among those who are especially prone to the disease are stone cutters; cigar makers and other workers in tobacco; plasterers; compositors, printers and pressmen; servants; hat and cap makers; bookkeepers, clerks and copyists; nonagricultural laborers; workers in tin; cabinet makers and upholsterers; musicians; glass blowers; barbers; coal miners,

1. Osler's Practice.

and other similar workers. In these occupations, we find the individual either exposed to fine, irritating dust, as in the case of the stone cutters, tobacco workers, hat makers, grain shovelers, upholsterers, and barbers, or exposed to bad air, cramped positions, and the like, as in the case of printers, clerks, and other indoor workers.

From this statistical report of Lillian Brandt, we further learn that "from the registration of living cases and from the records of death, not only is consumption more prevalent in certain parts of the city than in others, but that in any given district it is concentrated in certain streets, blocks and even houses. There are houses in which cases of consumption have occurred in each of the last nine years; and there are others in the same block from which none have been reported."

A careful study has been made by Dr. Flick of "the distribution of the deaths from tuberculosis in a single city ward in Philadelphia for twenty-five years. His researches go far to show that it is a house disease. About 33 per cent. of infected houses have had more than one case. Less than one-third of the houses of the ward became infected with tuberculosis during the twenty-five years prior to 1888. Yet more than one-half of the deaths from this disease during the year 1888 occurred in those infected houses."

Similar experiences could be multiplied indefinitely from the practice of all physicians; but enough has been said to illustrate thoroughly the infectious nature of the disease, and to impress upon us also the fact that, besides the presence of the bacillus, usually one or more other conditions are found as causal factors in any given case of consumption; but that without the bacillus no case could exist.

The infectious material is carried in the form of dust in the air, and is inhaled or swallowed. In the dejecta of the common housefly, tubercle bacilli have been found. These pests light on our food, and may thus be the means of carrying infection. It may be conveyed through the milk of tuberculous cattle, or, possibly, through meat of tuberculous animals,—though this is not very probable, as most meats are cooked before eaten. Anything that reduces the resisting power of the individual to disease in general renders him so much the more susceptible to the onslaught of consumption.

The children of sick parents are naturally not as strong as the children of healthy parents. In this respect only has heredity any bearing on the production of this disease. On the other hand, the environment in the way of occupation and home surroundings, has been shown to have a distinct bearing on the subject. Moreover, bad habits,—especially the excessive use of alcoholic stimulants and the frequenting of brothels,—have a distinct effect in lessening the resisting power of the individual to the onslaught of the disease. Briefly, we may say that darkness, dampness, bad air, bad habits and poor and insufficient food are the chief factors in the preparation of the soil for the implantation of the tubercle bacillus, and for the continuation of its evolution and growth afterward.

In the fight against this disease, then, everything should be done to improve the sanitary condition of the working places, so that there may be plenty of fresh air for each individual; that there be working space enough, that he does not have to take a cramped position,—or, if that is necessary for his work, that there be sufficient shifts of men that each individual may have a period of time for good lung expansion; that in dusty occupations, means be used to reduce the dust to a minimum, and that shields for the mouth and nose be provided for the workmen; that no one be allowed to drink alcoholic beverages during working hours; and that the existing tenement laws be strictly enforced and sufficient number of sanitary inspectors provided to make it possible to do the work thoroughly.

After a case of tuberculosis has left a dwelling,—either for a hospital or for a grave,—the room or rooms which he has occupied should be most thoroughly disinfected, and such articles of clothing or furniture as cannot be disinfected should be burned. The bodies of all dead of any infectious disease—including, of course, consumption—should be cremated. Sunlight, pure air and good food, all three in plentiful amount, are the chief factors in both the prevention and cure of tuberculosis; all efforts should be made to secure them for everyone in their homes and in their working places.

Practically, we must recognise the fact that the tubercular individual is with us, and we must concentrate a considerable amount of our effort in educating him how to avoid the infecting of his surroundings.

It has been well remarked by Cornet, "The consumptive in himself is almost harmless, and only becomes harmful through bad habits."

The expired breath of consumptives does not contain the bacilli; neither do the excretions from the skin, the kidney, or the bowel, unless there is ulcerative tubercular disease of these organs. The only source of danger in a consumptive is the sputum, finely divided particles of which are discharged during the act of coughing and are suspended in the atmosphere, thus infecting the surrounding air.

James B. Russell has well said:

> In order to be air-borne, the sputum must be dried and broken up into dust. If discharged into a handkerchief, it speedily dries, especially if put into the pocket or beneath the pillow. In the last stages of consumption, the patient becomes weak, the sputum is expelled imperfectly, pillows, sheets and handkerchiefs are soiled. If a male, the beard or moustache is smeared. Even in the hands of the cleanly, unless special precautions are taken, such circumstances tend to the production around the patient of a halo of infected dust maintained by every process of bed-making or of cleaning, which includes the pernicious process happily described as "dusting." In the hands of the careless and dirty, the infectivity is, of course, greatly aggravated. It attains its maximum of intensity where the filthy habit of spitting on the floor prevails.

If, however, proper care is taken of the sputum, there is no danger of infection from a consumptive, as has been thoroughly proven by experience of many sanatoria, the statistics from some of which I have given you.

What are the precautions that should be adopted? They are very few, and very simple—namely:

(1) The consumptive should kiss no one; and no one should kiss the consumptive on the mouth.

(2) The consumptive should never lend his handkerchief to anyone, or borrow a handkerchief from anyone.

(3) The eating and drinking utensils of a consumptive should be thoroughly boiled after he has used them.

(4) During the act of coughing, he should hold before his mouth a piece of cheesecloth, or gauze, or other material, which may be burned or boiled.

(5) The sputum should be discharged into a piece of cheesecloth or gauze, so folded that the sputum may be turned in and completely covered; such piece of gauze should be put into a proper receptacle, until it is time to take it out and burn it, and boil the receptacle. Similar pieces of gauze should be used for blowing the nose. These pieces of gauze can be burned once a day or oftener, according to their number.

(6) The sheets, pillow cases, night clothes and underclothing of consumptives should be boiled for half an hour before being washed.

If these simple precautions are taken, and heed is given to the injunction of Dr. John H. Pryor, "to care for the consumptive in the right place, in the right way, and at the right time until he is cured; instead of in the wrong place, in the wrong way, at the wrong time until he is dead," we need have little fear but that the disease will be wiped out of existence.

These precautions sound simple, and it seems that it should be necessary only to call attention to them, carefully explaining the reasons for them, to have them readily complied with. As a matter of fact, however, this is not true; and, in my opinion, we will never be rid of consumption until government control of the consumptive is an established fact and thoroughly carried out by those in authority.

From a long period of observation in connection with my attendance at the Erie County Hospital for tuberculosis, and in the ordinary course of a practice which brings under my observation many cases of pulmonary tuberculosis, I have become convinced that the chief cause of the persistence of tuberculosis and the main source of infection is the sputum of the chronic case who is able to be up and about and to do some work, but who is unable, or unwilling, to care properly for his sputum. In the autumn or winter these chronic cases come into the hospital, are well-fed, clothed and given abundance of pure air, and when the early summer comes they are in good enough physical condition to leave the institution, go out and go to work on the docks or elsewhere, occupy some room in a tenement, or lodge with the family of some friend, or occupy a bed in some cheap lodging-house,—often a different bed, and sometimes a different house every night. They pay no attention to the instructions they have received in regard to care of sputum, because they are their own masters and do not have to obey rules outside. Thus they cough and spit and scatter bacilli by the billions, infecting their surroundings wherever they go. If they feel depressed from any reason,—disease, fatigue or lack of food,—they usually have recourse to alcohol in some form, thus further depressing their resisting powers and making themselves even more careless and vicious in their habits.

Under the circumstances it seems clear that great good could be accomplished by the establishment in every community of two sanatoriums, one for advanced, and one for incipient, cases of pulmonary tuberculosis. By the enactment of an ordinance, every person with pulmonary tuberculosis who cannot receive proper care and be accommodated with an airy room at home to be occupied by himself alone, should be compelled to go to one or the other sanatorium.

Two sanatoriums are advocated: (1) because the treatment of the two classes of patients is decidedly different; (2) because the constant presence of advanced patients and the occasional painful death of one or more have a decidedly depressing influence upon those having incipient disease. That this is a real influence for the bad, I have had demonstrated on several occasions in a mixed institution with which I have been connected for several years. One winter two patients that were regularly and steadily improving in all respects, took a sudden and decided change for the worse after distressing deaths of other patients in an advanced stage of the disease.

If every case of pulmonary tuberculosis were reported to the health department, as it should be, the department could send an inspector and determine whether such case is one for home or sanatorium treatment. Of course every patient should be allowed the privilege of sanatorium treatment if he so desire, even though his circumstances were such that he could be cared for at home. The health authorities are allowed to isolate cases of smallpox and certain other infectious diseases, and the same right should be accorded them with regard to the greatest destroyer of all—tuberculosis.

After removal of the patient, his bedding, clothing and apartment should be thoroughly disinfected and cleaned. In this way many foci for the distribution of the disease could be destroyed. In the sanatorium the patient would, of course, be under strict medical supervision so that slight disturbance of temperature and other indications of increase in virulency of the disease, even though slight, would be noted early and thus there would be afforded a much better chance to arrest its progress by the prompt establishment of proper therapeutic measures than if the patient were at home under only occasional medical observation. In this way the chances for the patient's recovery are doubled and he is no longer a source of danger to his fellows.

Moreover, in the sanatorium, he learns, through observation as well as instruction, how to care for his sputum and the very great value of this simple procedure. Through his visits to his friends and their visits to him, this knowledge would become much more widespread than by all the books and public lectures put together. Daily observation of the thing done acts as a clinical lecture, leaving a vivid impression much more lasting than that produced by the best of didactic instruction.

It will be urged that many tuberculous persons are able to attend to business and aid in the support of themselves and their families. To this it may be replied that it will be much less expensive to quit work for six months, or even for eighteen months and get well, than to continue doing rather poor work for a few months or a year, and then have to quit for all time. Moreover, sanatorium life need not interfere with business, if the patient really is in condition to attend to business, but it will interfere when it is best for himself and his companions that it should do so. This is, in truth, no hardship, but ultimately a gain for all.

It may also be urged that it is an unnecessary hardship to compel a patient to die in an institution, excluding him from the privilege of passing his last days in the bosom of his family. To a very limited degree this is true, but in tuberculosis the end is seldom sudden and the members of the family can be notified and allowed to be present at such a time.

In the case of other great plagues, the isolation is more complete, and the friends are not allowed to be present even at the last. So the procedure is not unprecedented, and the good to come to the entire race from such sequestration, kept up persistently for 10 or 15 years, would far outweigh the slight psychic disturbance of a few. The bodies of all dead from tuberculosis should of course be cremated.

Instruction in regard to destruction of sputum and feces, in regard to precaution as to personal contact with others and in regard to disinfection of apartments lately occupied by the tuberculous, are all of value in checking the spread of the disease; but it is my opinion that some such plan as has been outlined, of compulsory reporting of cases and compulsory removal of suitable patients to their proper sanatoriums, will be much more efficacious than all the other procedures in ridding us of the great white plague.

469 Franklin Street.

